# Priority effects can be explained by competitive traits

**DOI:** 10.1002/ecy.4528

**Published:** 2025-01-21

**Authors:** Tamara L. H. van Steijn, Paul Kardol, Roland Jansson, Jessica Tjäder, Judith M. Sarneel

**Affiliations:** ^1^ Department of Ecology and Environmental Science Umeå University Umeå Sweden; ^2^ Department of Forest Mycology and Plant Pathology Swedish University of Agricultural Sciences Uppsala Sweden

**Keywords:** competition, competitive effect and response, functional similarity, plant functional groups, plant interaction, priority effects, traits

## Abstract

Priority effects, the effects of early‐arriving species on late‐arriving species, are caused by niche preemption and/or niche modification. The strength of priority effects can be determined by the extent of niche preemption and/or modification by the early‐arriving species; however, the strength of priority effects may also be influenced by the late‐arriving species, as some species may be better adapted to deal with niche preemption and/or modification. Therefore, some combinations of species will likely lead to stronger priority effects than others. We tested priority effects for all pairwise combinations of 15 plant species, including grasses, legumes, and nonleguminous forbs, by comparing simultaneous and sequential arrival orders in a 10‐week‐long, controlled, pot experiment. We did this by using the competitive effect and response framework, quantifying the ability to suppress a neighbor as the competitive effect and the ability to tolerate a neighbor as the competitive response. We found that when arriving simultaneously, species that caused strong competitive effects also had weaker competitive responses. When arriving sequentially, species that caused strong priority effects when arriving early also had weaker responses to priority effects when arriving late. Among plant functional groups, legumes had the weakest response to priority effects. We also measured plant functional traits related to the plant economic spectrum, which were combined into a principal components analysis (PCA) where the first axis represented a conservative‐to‐acquisitive trait gradient. Using the PCA species scores, we showed that both the traits of the focal and the neighboring species determined the outcome of competition. Trait dissimilarities between the focal and neighboring species were more important when species arrived sequentially than when species arrived simultaneously. Specifically, priority effects only became weaker when the late‐arriving species was more acquisitive than the early‐arriving species. Together, our findings show that traits and specifically the interaction of traits between species are more important in determining competition outcomes when species arrive sequentially (i.e., with priority effects present) than when arriving simultaneously.

## INTRODUCTION

Plants compete with their neighbors for resources such as light, water, and nutrients, and differences between the competitiveness of species lead to variation in the relative abundance of species in a plant community. The competitiveness and thus abundances of species might in turn change, for example, due to plant–soil feedbacks, weather fluctuations, and changes in climate (Allan et al., 2011). The competitive ability of a species has two sides: the ability to suppress a neighbor, referred to as competitive effect, and the ability to tolerate a neighbor, referred to as the competitive response (Goldberg, 1990). Competitive effect and response are often related, meaning that species that cause stronger competitive effects are also more tolerant to competition from neighbors themselves (Goldberg & Fleetwood, 1987; Rösch et al., 1997; Wang et al., 2010; Zhang & Lamb, 2012). However, the relationship between the two is not static and there is increasing evidence that competitive response often is more variable than competitive effect. For example, competitive response depends on life stage (Zhang & Lamb, 2012) and is influenced by environmental factors, such as soil fertility (Wang et al., 2010) or disturbances (Keddy et al., 1994). Therefore, Wang et al. (2010) suggested that competitive effect is a characteristic of a species, while competitive response is variable across environments and neighboring species.

Plant functional traits can explain the competitive ability of species. For example, a mechanism for aboveground competition is light acquisition, where fast‐growing plants are able to acquire much light due to having more leaf area, making it harder for neighboring plants to establish (Hautier et al., 2009). A mechanism for belowground competition is the acquisition of water and nutrients, where species with fast‐growing roots will have a competitive advantage over their neighbors (Fort et al., 2014). Furthermore, the differences in traits between species have been used to explain the outcome of competitive interactions. For example, Herben et al. (2020) showed that differences in ramet biomass, leaf area, and rooting depth between competing species correlated with competition strength. Further, Conti et al. (2018) used differences in height, specific leaf area (SLA), and seed mass between competing species to explain the biotic resistance of natives to invasives. A belowground example comes from Fort et al. (2014), who used differences in specific root length, root phosphorus use efficiency, and root length density to explain competition strength. It has been suggested that the traits of competing species influence species coexistence and that large differences in traits related to niche differentiation reduce the intensity of competition for the same resources, while traits related to competitive hierarchy should be similar, so that one species does not outcompete the other (Herben & Goldberg, 2014).

Priority effects can also dramatically alter the outcome of competitive interactions (Fukami, 2015). These effects, caused by variation in arrival order, can impact various aspects of plant communities, such as diversity (e.g., Dickson et al., 2012), productivity (e.g., Körner et al., 2008), and resilience to invasion (e.g., Vaughn & Young, 2015). Intuitively, the species that arrive early often benefit from the preemption of resources, giving them a competitive advantage over later‐arriving species. This is referred to as a negative priority effect (e.g., Grman & Suding, 2010; Ploughe et al., 2020). For example, grasses can cause negative belowground priority effects by dense shallow rooting, making it harder for late‐arriving plants to establish (Alonso‐Crespo, Temperton, et al., 2023; Alonso‐Crespo, Weidlich, et al., 2023). However, there is also evidence for positive, facilitative priority effects, probably through beneficial modification of the environment. For example, Delory, Weidlich, von Gillhaussen, et al. (2019) showed that sowing legumes before grasses and forbs had a positive effect on biodiversity after 1 year, likely due to legumes providing protection against invasion and because of their nitrogen‐fixing ability. Hence, plant functional groups are often used to test priority effects, assuming similarity of species within the same functional group. However, there is variation of traits within functional groups and they might therefore be too coarse for understanding how priority effects are influenced by plant functional traits.

Even though priority effects can drastically alter the outcome of competitive interactions, they have to our knowledge never been placed in the competitive effect and response framework. In other words, we do not know how priority effects influence the relationship between competitive effect and response. Furthermore, although several studies have tested the role of species traits in driving the strength of priority effects, especially by using plant functional groups (e.g., Delory, Weidlich, von Gillhaussen, et al., 2019; Helsen et al., 2016; Weidlich et al., 2018), we do not know how the *differences* in traits between early‐ and late‐arriving species influences the strength of priority effects. To bridge these knowledge gaps, we ran a growth chamber experiment with the objective to test how priority effects influence aboveground and belowground competitive effects and responses and how these competitive effects and responses are mediated by plant functional groups and traits. To do this, we grew all possible pairwise combinations of 15 grassland plant species, either planting them simultaneously or sequentially.

We tested the following hypotheses: (1) When species arrive early, they will have a stronger negative competitive *effect* than when arriving simultaneously. Further, when species arrive late, they will have a stronger negative competitive *response* than when arriving simultaneously. (2) Legumes cause weaker priority effects, while grasses cause stronger priority effects. This is because legumes fix nitrogen, leading to less nutrient‐depleted soils, while grasses have dense root growth, leading to strong belowground effects. (3) Plant functional traits related to the acquisitive‐conservative gradient can predict how species are affected by competition, both with and without priority effects present. Specifically, acquisitive species should have a stronger negative competitive effect and a less negative competitive response than conservative species. (4) Finally, the dissimilarity in traits related to the acquisitive‐conservative gradient is correlated to the strength of competition. Specifically, competition should be stronger when species are dissimilar, favoring the more acquisitive species. When priority effects are present, the relationship between trait dissimilarity and competition strength should become even stronger, since species will be dissimilar not only in traits but also in the opportunity for resource preemption.

## METHODS

**FIGURE 1:**
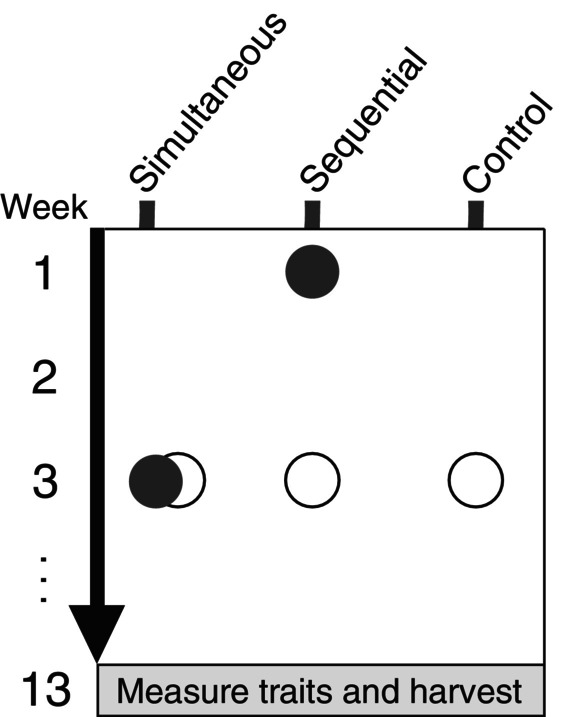
Illustration of the experimental setup depicting the different arrival orders. Each dot represents when seedlings were planted, with solid and open dots representing different species. Treatments were applied for all pairwise combinations of 15 species (*n* = 1). The controls were replicated five times per species.

We selected 15 grassland species from three functional groups (forbs, grasses, and legumes; Table 1). The species were chosen to cover a large range of traits and therefore competitive ability, from small, creeping forbs to large, fast‐growing grasses, and are commonly found in various types of meadows and grasslands. We grew these species in a growth chamber experiment where we applied three treatments (Figure 1). In the first treatment, we planted two species with one seedling each together in one pot and grew them for 10 weeks (simultaneous arrival, all pairwise combinations with one replicate each, 105 pots). In the second treatment, we planted seedlings of two species sequentially, with an interval of 3 weeks, and grew them for 10 weeks since the arrival of the second seedling (sequential arrival, all pairwise combinations with one replicate each, 210 pots). In the third treatment, we planted one seedling of each species alone in one pot and grew them for 10 weeks (controls, five replicates per species, 75 pots).

**TABLE 1:** Plant species used in the experiment, their functional group, plant growth form, and seed vendor (see footnote).

Species	Functional group	Plant growth form
*Agrostis capillaris* (L.)^a^	Grass	Tufted with creeping rhizomes
*Antennaria dioica* (L. Gaertn.)^b^	Forb	Ground‐hugging and mat‐forming
*Briza media* (L.)^b^	Grass	Tuft‐forming
*Cardamine pratensis* (L.)^b^	Forb	Basal rosette
*Dianthus deltoides* (L.)^b^	Forb	Loosely tufted and mat‐forming
*Festuca rubra* (L.)^a^	Grass	Tuft‐forming
*Lathyrus sylvestris* (L.)^a^	Legume	Climbing
*Leucanthemum vulgare* (Lam.)^b^	Forb	Creeping rhizomes
*Lotus corniculatus* (L.)^b^	Legume	Tap rooted
*Phalaris arundinacea* (L.)^b^	Grass	Tall with rhizomes
*Poa pratensis* (L.)^a^	Grass	Creeping rhizomes
*Sedum acre* (L.)^b^	Forb	Low growing, shallow rooted and creeping
*Trifolium pratense* (L.)^a^	Legume	Tap rooted
*Trifolium repens* (L.)^b^	Legume	Mat‐forming through stolons
*Vicia cracca* (L.)^a^	Legume	Fast growing and climbing

*Note*: All seeds were cultivated, with wild populations in the ancestral line.

^a^ Cruydt Hoeck (Nijeberkoop, NL).

^b^ Jelitto Staudensamen (Schwarmstedt, DE).

### Experimental setup

Plastic pots with a diameter of 13 cm and a height of 10.5 cm were filled with 2 L of a mixture of two parts potting soil (NPK 14‐7‐15; Kekkilä‐BVB, Vantaa, Finland) and one part sand (grout sand, Bygmaxx, Solna, Sweden). Prior to the start of the experiment, we made five soil extracts of the soil mixture and analyzed them for pH and nutrient concentrations (Appendix S1: Section S1.1). Plastic cones (clear filter no. 130; Lee, Hampshire, UK) were wrapped around the pots to prevent plants from neighboring pots from interfering with each other (Appendix S1: Figure S1). Seeds were obtained from vendors (Table 1). Each species was germinated in separate trays in the same soil mixture as used in the main experiment. Sowing was carried out in phases, and seedlings at the stage of the emergence of the first true leaves were selected for all species in all treatments.

In simultaneous and sequential treatments, seedlings were planted 2 cm from the center of the pot and at opposite ends (hence 4 cm between them). Seedlings that died within the first 2 days of planting were replaced. If plants died after that, we discarded the pot and started a new one. However, five of 390 pots were discarded because of plant death past the halfway point of the experiment.

Pots were placed in trays (six per tray) and bottom‐watered twice per week to prevent soil moisture from being a growth‐limiting factor. Weekly, we randomized the position of trays in the growth chamber to minimize effects of slight variations in growing conditions. The temperature of the growth chamber was programmed with a day–night cycle of 22/18°C. Lights of the growth chamber had a sun‐like spectrum (252 ± 19 SD μmol m^−2^ s^−1^ at soil surface level; Valoya, Helsinki, Finland), with a day–night cycle of 16/8 h.

After 10 weeks (for the sequential treatment after 13 weeks), all pots were harvested to determine aboveground and belowground dry biomass (Figure 1). Aboveground biomass was clipped at the soil level. Roots were washed and, for plants grown in two‐species mixtures, carefully separated. All biomass samples were dried at 60°C for at least 48 h.

### Trait measurements

At the time of harvest, the following traits were determined for the plants in the control treatment. Absolute growth rate was calculated as a measure of productivity by dividing final total biomass by the number of growing days (AGR, per gram per day). We used absolute instead of relative growth rate, since the starting masses of the seedlings were negligible compared with their final masses. Root mass fraction (RMF) was calculated as the fraction of belowground dry biomass of total dry biomass. Plant height (in millimeters) was measured as the distance from the soil to the highest point of the plant. Lateral spread was measured as the maximum distance between shoots (in millimeters), which was restrained by the pot diameter of 13 cm. Ten leaves per plant were harvested and scanned to calculate the mean leaf area (LA, in square millimeters) using the software Fiji (Schindelin et al., 2012) after which the total fresh and dry weight were determined. Leaf dry matter content (LDMC) was calculated as the ratio of leaf dry mass to fresh mass. SLA (in square meters per kilogram) was calculated as the ratio of leaf area to leaf dry mass.

### Relative Intensity Index

To quantify competitive effects and responses across treatments, we calculated Relative Intensity Index (RII) (Armas et al., 2004) for each individual in each pair of species. In short, RII is constrained between +1 and −1, where a value of +1 implies that the focal species experiences facilitation by the neighboring species, a value of 0 implies that the focal species is unaffected by the neighboring species, and a value of −1 implies that the focal species is completely outcompeted by the neighboring species. RII_sim_ was calculated for each species combination after simultaneous arrival as:

(1) $$\mathrm{RI}I_{\mathrm{sim}\left(a,b\right)}=\left(M_{a}-M_{a+b}\right)/\left(M_{a}+M_{a+b}\right).$$

**FIGURE 2:**
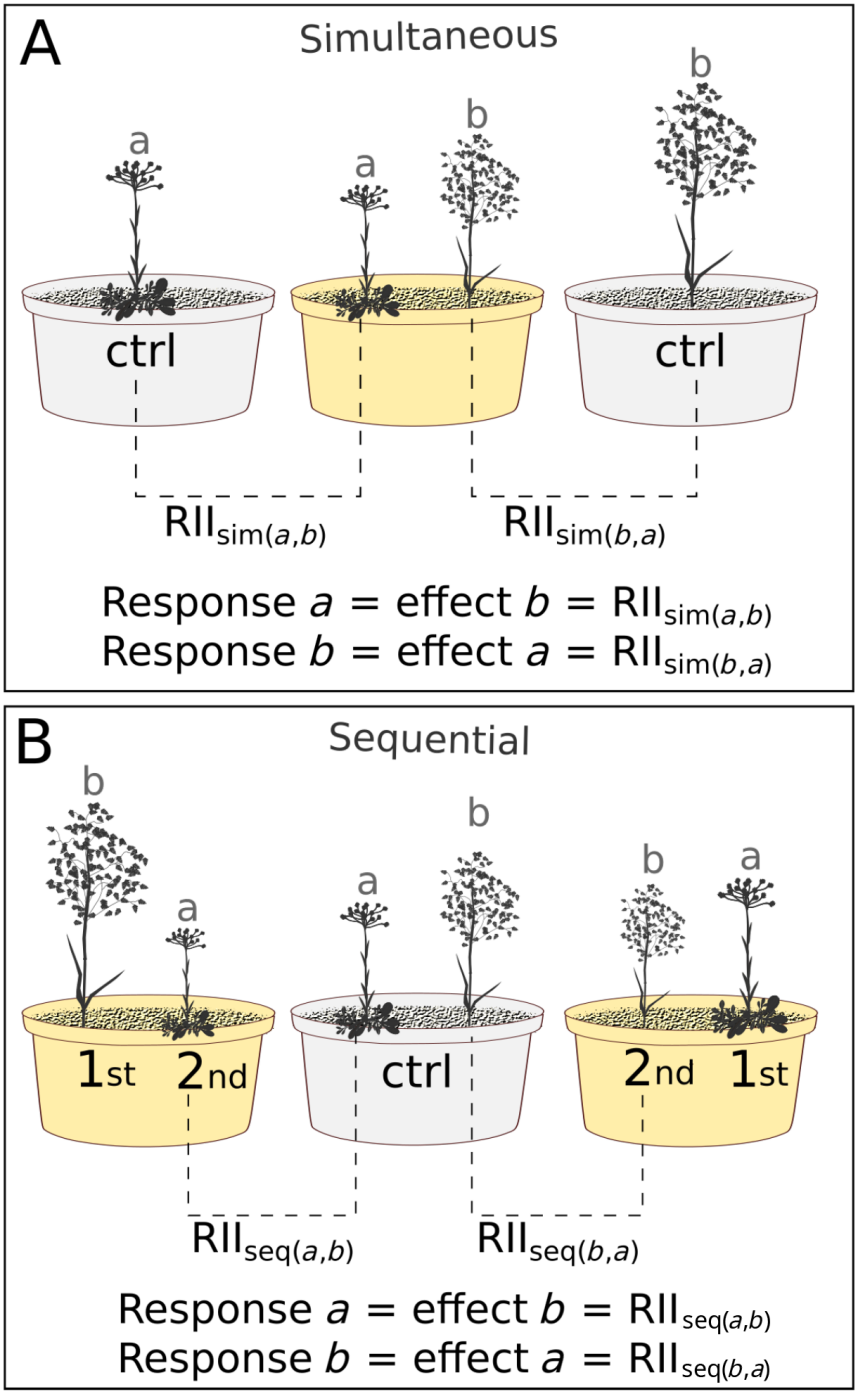
Visualization of the calculation of the competitive effect and response using the Relative Intensity Index (RII_sim_ [panel A] and RII_seq_ [panel B]) using *Antennaria dioica* (species a) and *Briza media* (species b) as examples. Pots with plants for which RII is calculated are indicated in yellow. In panel B, the numbers indicate the order of arrival. Note that the treatment of RII_sim_ becomes the control for RII_seq_. Illustration credits: Tamara L. H. van Steijn.

In Equation (1), *M*_*a*_ is the biomass of species *a* grown alone and *M*_*a*+*b*_ is the biomass of species *a* when grown together with species *b*. RII_sim(*a*,*b*)_ is the competitive response of *a*, which will henceforth be referred to as the focal species, while also being the competitive effect of *b*, which will henceforth be referred to as the neighboring species (Figure 2A). We focused on intraspecific competition, and we included competition for space in the pot as part of RII_sim_. Likewise, we calculated RII_seq_ for each species combination after sequential arrival as:

(2) $$\mathrm{RI}I_{\mathrm{seq}\left(a,b\right)}=\left(M_{a+b}-M_{b>a}\right)/\left(M_{a+b}+M_{b>a}\right).$$

In Equation (2), *M*_*a*+*b*_ is the biomass of species *a* grown together with species *b* and *M*_*b*>*a*_ is the biomass of species *a* when arriving after species *b*. For RII_seq(*a*,*b*)_, the late‐arriving species *a* is referred to as the focal species, while the early‐arriving species *b* is referred to as the neighboring species. Generally, priority effects are considered the effects of early‐arriving species on late‐arriving species; therefore, we only considered the effect of arriving late and not of arriving early (Figure 2B). To test for differences in aboveground and belowground competition (henceforth referred to as competition space), RII_sim_ and RII_seq_ were calculated for both aboveground and belowground biomass. All biomass values used in the calculations were from plants that were 10 weeks old; therefore, there is no bias in the results that stems from comparing plants of different ages (Figure 1).

We chose an approach of maximizing the number of species used in the experiment but with low replication (*n* = 1 per species pair) as opposed to using fewer species with higher replication. For the competitive response and effect analysis and for the functional group analysis, we averaged the species RIIs at either the species (*n* = 15) or functional group (*n* = 5) level, respectively. Importantly, by using a higher number of species as opposed to a high number of replicates, we captured a wider range of traits. This allowed us to use trait values on a continuous scale to study both the effects of traits of the focal species, traits of the neighboring species, and trait dissimilarity on RII.

### Statistics

To test whether priority effects led to stronger negative competitive effects and responses, we compared RII_sim_ and RII_seq_ using paired *t* tests with pairs based on unique species combinations. We used linear models to test if the average competitive response (independent factor) of a species was related to its average competitive effect (dependent factor). We included a quadratic term for competitive response when this was found to significantly improve the model, which we determined by model comparisons using ANOVA (R Core Team, 2023).

To evaluate the importance of plant functional traits on the outcomes of competitive interactions, we first tested whether RII_sim_ and RII_seq_ were affected by the functional group of the focal species, the functional group of the neighboring species, and competition space (i.e., whether the RII was calculated using aboveground or belowground biomass). Further, we tested interactions between those variables to see whether certain functional groups had stronger aboveground or belowground competitive effects or responses on or to other functional groups. To do this, we used two three‐way ANOVAs (R Core Team, 2023), one for RII_sim_ and another for RII_seq_. We used Tukey's honestly significant difference (HSD) post hoc tests to test for differences between the group means if the ANOVA indicated them. Second, we ran a principal components analysis (PCA) to reduce the dimensionality of the plant trait data (Vegan package, Dixon, 2003).

We extracted species scores along the first two axes and interpreted them as integrative measures of plant strategies. We then fitted linear mixed‐effect models (LME4 package, Bates et al., 2015) to test whether plant functional traits explained RII, fitting one model for RII_sim_ and one for RII_seq_. For both models, the fixed effects were PCA axis scores of the focal species, the PCA axis scores of the neighboring species, competition space, and all possible interaction terms. We added the species identities of both focal and neighboring species and pot number as separate random effects.

The interaction between traits of the focal species and neighboring species was further explored by calculating trait dissimilarity between competing species. To do this, we subtracted the PCA axis scores of the neighboring species from those of the focal species, separately for both PC1 and PC2.

Values around zero indicate that species have similar traits, and larger negative or positive values indicate larger trait dissimilarities between competing species. We then used the values of trait dissimilarity in linear mixed‐effects models with RII_sim_ and RII_seq_ for aboveground and belowground biomass, using the general approach described above. We tested for nonlinearity by adding quadratic terms for trait dissimilarity and kept them in the model if it significantly improved the model which was tested using ANOVA.

## RESULTS

Priority effects were common and strong. When species arrived sequentially, late‐arriving plants had on average 73% ± 29 SD less biomass than plants that arrived simultaneously (Appendix S1: Figure S2). The average RII_seq_ was significantly more negative than the average RII_sim_ (−0.73 ± 0.29 SD and −0.26 ± 0.24 SD, resp., paired *t* test: *t* = −21.1, df = 201, *p* < 0.001), indicating that plants arriving second experienced stronger competition than plants arriving simultaneously with their neighbor.

### Competitive effect and response

**FIGURE 3:**
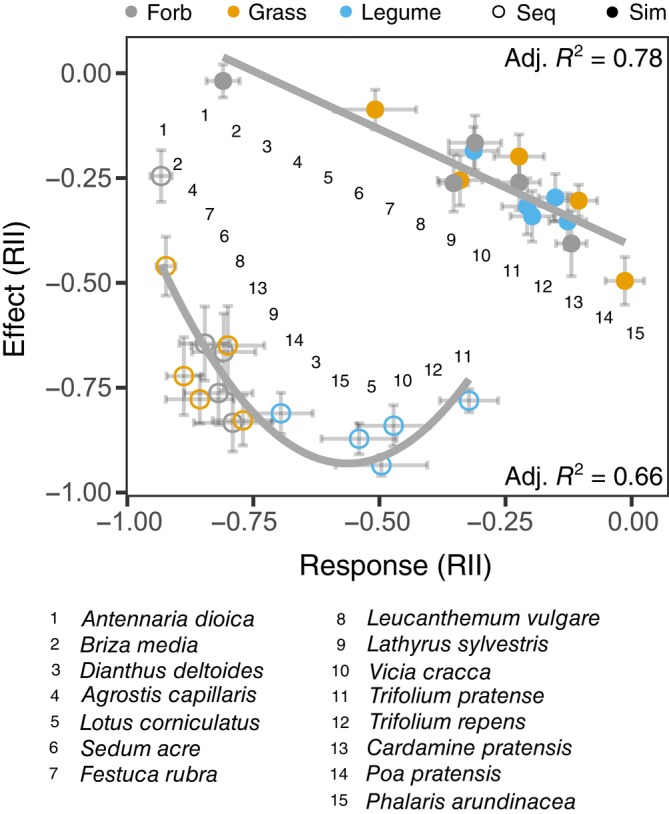
The relationships between the Relative Intensity Indices (RIIs) of competitive effect and competitive response when species arrived simultaneously (sim, filled symbols) and sequentially (seq, open symbols). Each dot is the average of a species competitive effect and response with SEs. These averages are calculated for each species when competing against each of the other species; therefore, *n* for each dot is 14. Large negative competitive effects indicate that a species has a strong impact *on* its neighbor, while a large negative competitive response indicates that a species is strongly impacted *by* its neighbor. Adjusted *R*‐squared values are displayed for significant models (Appendix S1: Tables S2 and S3). The numbers in the plot indicate the order of species along the curves.

We found that the competitive effect of species significantly predicted their competitive response, following a linear relationship for simultaneous arrival (RII_sim_, df = 13, *F*‐statistic = 49.67, Adj. *R* ^2^ = 0.78, *p* < 0.001) and a quadratic relationship for sequential arrival (RII_seq_, df = 12, *F*‐statistic = 14.34, Adj. *R* ^2^ = 0.66, *p* = 0.001; Figure 3; Appendix S1: Section S1.4). In other words, species that caused large negative effects on their neighbors were less impacted by competition themselves. For RII_seq_, the relationship rapidly approached –1, which is the lower limit of RII_seq_.

In addition to the difference in shape of the relationship between competitive effect and response when arriving simultaneously versus sequentially, the order of species along the curves also changed. This indicates that the interaction between species when they arrive simultaneously does not predict how they interact when arriving sequentially. For RII_sim_, the species with the strongest effects and weakest responses were a mixture of grasses, forbs, and legumes, while for RII_seq_, they were all legumes, indicating that legumes cause both stronger priority effects when arriving first, while also being less impacted by priority effects when arriving second (Figure 3).

### Plant functional groups

When species arrived simultaneously, belowground competition was on average stronger than aboveground competition (df = 1, *F*‐value = 5.68, *p* = 0.018). A Tukey HSD post hoc test showed that within functional groups, this difference was only significant for grasses. Further, forbs had a significantly stronger negative competitive response than the other functional groups (df = 2, *F*‐value = 24.53, *p* < 0.001). We found an interaction between the functional group of the focal species and the functional group of the neighboring species; for legumes, we found that they had a significantly stronger negative competitive response to other legumes (df = 4, *F*‐value = 2.36, *p* = 0.053; Appendix S1: Section S1.5).

**FIGURE 4:**
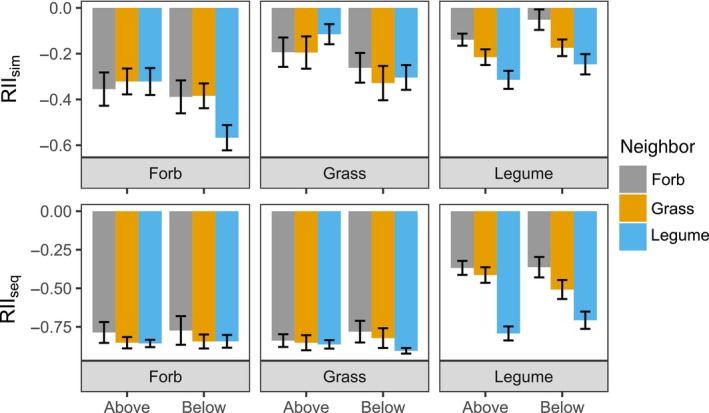
Differences in the Relative Intensity Index (RII), both when arriving simultaneously and sequentially (RII_sim_ and RII_seq_), aboveground and belowground for forbs, grasses, and legumes. The focal species belong to the functional groups displayed on the plot axis, for which RII is the competitive response. The neighboring species belong to the functional group according to the color coding, for which RII is the competitive effect. For RII_seq_, the neighbor is the species that arrives early, while the focal is the species that arrives late. Error bars are SE (mean number of pots per bar = 23.1 ± 2.5 SD). For ANOVA and Tukey honestly significant difference (HSD), see tables in Appendix S1: Section S1.5.

When species arrived sequentially, we found that legumes were less negatively impacted by priority effects (df = 2, *F*‐value = 81.99, *p* < 0.001). Forbs and grasses had weaker competitive effects than legumes (df = 2, *F*‐value = 16.91, *p* < 0.001). These results led to a significant interaction between the functional group of the focal and neighboring species; legumes were on average less negatively impacted by priority effects when the early‐arriving species was either a grass or a forb (df = 4, *F*‐value = 7.06, *p* < 0.001). There were no significant differences in priority effects due to competition space (i.e., aboveground vs. belowground competition, df = 1, *F*‐value = 0.04, *p* = 0.838; Figure 4; Appendix S1: Section S1.5).

### Functional traits

**FIGURE 5:**
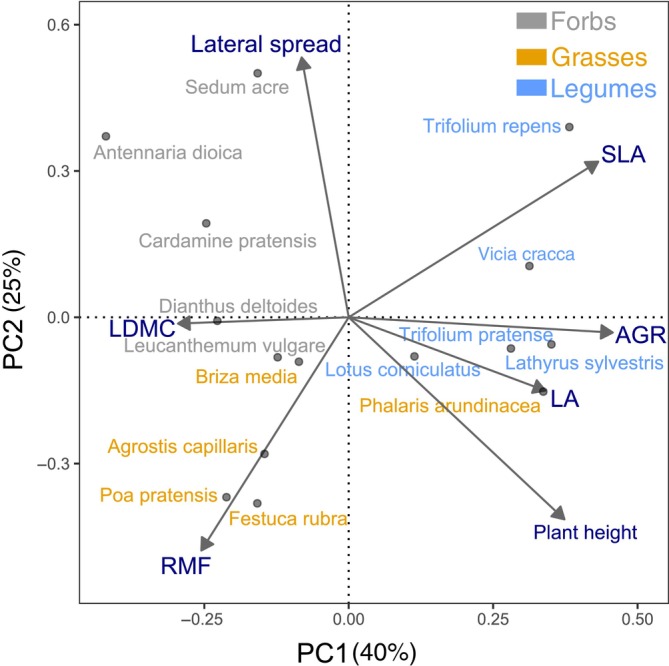
Principal components analysis (PCA) integrating the seven plant functional traits measured on the control plants across the 15 species (*n* = 5 per species). Arrows indicate the direction and strength of the correlation of the traits with the PCA axis. The measured traits are root mass fraction (RMF), leaf dry matter content (LDMC), leaf area (LA), specific leaf area (SLA), average plant height, clonal spread, and biomass and growth rate (AGR). Axes are interpreted to represent different strategies; the PC1 axis corresponds with the fast–slow spectrum, with conservative species scoring low and acquisitive species scoring high. The PC2 axis represents differences in plant form and stature, with species that invest less in roots but more in lateral spread scoring high and vice versa for species scoring low.

The first and second axes of a PCA of the plant traits explained respectively 40% and 25% of the total variance of the species trait values (Figure 5). The first axis (PC1) correlated negatively with LDMC and positively with growth rate and leaf area, and to a lesser extent plant height and SLA. We interpreted the PC1 axis as to the plant economic spectrum, where species with low scores have a conservative strategy and species with high scores have an acquisitive strategy (Reich, 2014). The second axis (PC2) correlated positively with lateral spread and SLA and negatively with RMF and, to a lesser extent, average plant height. We interpreted the PC2 axis as an axis of plant growth form and stature, where species with high scores are small, horizontally spreading plants with high SLA and a relatively low investment in roots.

**FIGURE 6:**
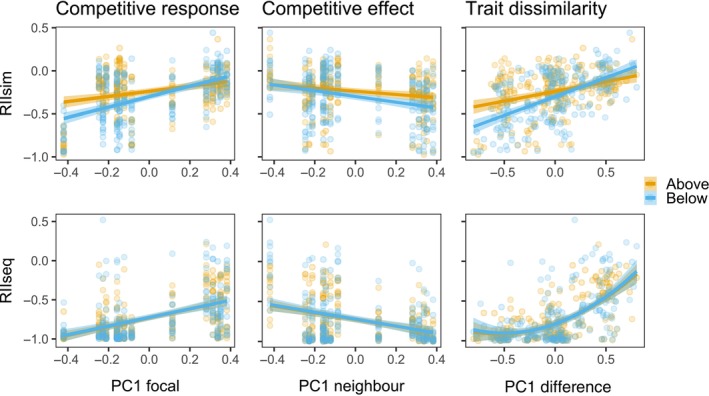
Relationships between PC1 species scores and the Relative Intensity Indices (RIIs), which are calculated based on aboveground and belowground biomasses for RII_sim_ (top panels) and RII_seq_ (bottom panels). RII values of around zero indicate no effect of competition, while values close to –1 indicate strong competition. Each dot represents one pot. Positive relationships in the left‐hand panels indicate that species with high PC1 scores have smaller competitive responses. Negative relationships in the middle panels indicate that species with high PC1 scores have larger competitive effects. Positive relationships in the right‐hand panels indicate that when the focal species had high and the neighboring species had low PC1 scores, the negative effects of competition become smaller (i.e., approach zero). Note that for RII_seq_, the focal species is the late‐arriving species, while the neighboring species is the early‐arriving species.

When arriving simultaneously, we found significant main effects of the PC1 score on the RII of the focal (*F*(1,12.97) = 8.19, *p* = 0.013) and neighboring species (*F*(1,12.99) = 7.13, *p* = 0.019, Figure 6). We further found a significant main effect of competition space when species arrived simultaneously (*F*(1,379.35) = 11.51, *p* < 0.001). We found significant interactions between competition space and the PC1 score of the focal species (*F*(1,379.35) = 17.92, *p* < 0.001), but not between the PC1 score of the neighboring species (*F*(1,379.35) = 3.19, *p* = 0.075). We did not find an interaction between the PC1 score of the focal species and the PC1 score of the neighboring species (*F*(1,380.18) = 1.19, *p* = 0.276). The full model for RII_sim_ had a marginal *R* ^2^ of 0.25 (Appendix S1: Table S10).

When arriving sequentially, we also found significant main effects of the PC1 score on the RII of the focal (*F*(1,12.99) = 20.55, *p* < 0.001) and neighboring species (*F*(1,12.99) = 7.44, *p* = 0.017). We did not find a main effect of competition space (*F*(1,283.16) = 0.10, *p* = 0.758). We found a significant interaction between the PC1 score of the focal and neighboring species when species arrived sequentially (*F*(1,84.52) = 0.74, *p* = 0.002). The full model for RII_seq_ had a marginal *R* ^2^ of 0.33 (Appendix S1: Table S11).

We explored the interaction between traits of the focal species and traits of the neighboring species further using trait dissimilarities, that is, the difference in PCA axis scores of the focal and neighboring species. We found that trait dissimilarities significantly predicted the outcomes of competitive interactions both when species arrived simultaneously (*F*(1,20.15) = 13.62, *p* = 0.001, full model marginal *R* ^2^ = 0.17, Appendix S1: Table S12) and sequentially (*F*(1,37.06) = 22.09, *p* < 0.001, full model marginal *R* ^2^ = 0.35, Appendix S1: Table S13). We repeated the analysis for the PC2 axis scores but did not find any significant relationships with RII_sim_ or RII_seq_ (Appendix S1: Section S1.7).

## DISCUSSION

### Competitive effect and response

Our results show that in support of our first hypothesis, both competitive effect and response became stronger when priority effects were present. That is, when species arrived simultaneously, species that caused stronger competitive effects on their neighbors were also less impacted by competition themselves, following a linear relationship. With the inclusion of priority effects, the relationship between competitive effect (i.e., the priority effect of the early‐arriving species on the late‐arriving species) and competitive response (i.e., the response of the late‐arriving species to the priority effect) became steeper and nonlinear. Here, the competitive response rapidly approached –1, showing that priority effects generally intensify competition, to a point where the late‐arriving species are almost completely outcompeted.

We also found a shift in the order of species along the competitive effect–response curves between simultaneous and sequential arrival, indicating a change in the competitive hierarchy within the species pool. Specifically, when species arrived simultaneously, the strongest competitors (i.e., those with large competitive effects and small competitive responses) included a mixture of forbs, grasses, and legumes. However, when species arrived sequentially, the strongest competitors were all legumes. Our functional group analysis illustrated that this shift in hierarchy resulted from weaker priority effects experienced by legumes when the first‐arriving species belonged to a different functional group.

This finding is important as it not only shows that competitive effects and responses are amplified when species arrive sequentially but also highlights a shift in the competitive hierarchy due to the presence of priority effects. These insights contribute to our understanding of how priority effects can alter community composition. For example, when species arrive simultaneously, a native species might outcompete an invasive species. However, when invasive species arrive or emerge earlier in the growing season, priority effects caused by invasive species could be strong enough to suppress the otherwise superior native species (Ulrich & Perkins, 2014). Furthermore, in the context of ecological restoration, this knowledge can be applied by giving priority to native species. Actively manipulating the order of species arrival through seeding to give priority to native species could increase their competitive ability, making restored communities more resilient to invasion (Hess et al., 2019).

### Aboveground and belowground competition

When species arrived simultaneously, belowground competition was on average stronger than aboveground competition, in particular for grasses, which supports our second hypothesis. This is likely because grasses tended to invest more in roots (high RMF; Figure 5) than other functional groups. A larger investment in root mass could also indicate more efficient nutrient uptake and sustained aboveground growth, leading to smaller competitive responses. However, when species arrived sequentially, aboveground and belowground competitions were equally strong. Most species remained smaller when arriving second, and the RII_seq_ was overall substantially more negative than the RII_sim_. In other words, when plants arrived late, they survived, but both aboveground and belowground growth was severely hampered. However, our experiment might not have been able to parse out more subtle belowground effects as we measured belowground dry biomass, but not root architecture or other root traits. For example, Alonso‐Crespo, Temperton, et al. (2023) and Alonso‐Crespo, Weidlich, et al. (2023) found that the arrival order of plant functional groups could affect vertical root distribution, which was altered when grasses arrived early. In all, our findings show that the strength of aboveground and belowground priority effects was comparable in both strength and direction.

### Plant functional groups

When species arrived simultaneously, we found that forbs are more affected by competition than grasses and legumes, likely because the forbs in our species pool tended to adhere to a more conservative strategy. In contradiction to our second hypothesis, we did not find that legumes caused smaller priority effects. However, we did find that legumes were on average less impacted by priority effects than the other functional groups. It has been shown that when nonleguminous plants are grown next to legumes, legumes increase their rate of nitrogen fixation (Carlsson et al., 2009). This mechanism has long been applied in agriculture to increase soil nitrogen through intercropping legumes with cereals (e.g., Hauggaard‐Nielsen et al., 2008). The legumes in our experiment may have been less affected by nitrogen‐depleted soils, therefore being less affected by priority effects.

Further, we found that legumes were primarily negatively affected by other legumes. It has been shown that high legume abundances can decrease the nitrogen‐fixing ability of co‐occurring legumes (Carlsson et al., 2009). The process of carbon deposition in the soil by roots (rhizosphere priming) may have contributed to our results. Rhizosphere priming leads to faster decomposition of soil organic matter and is a more energy‐efficient way of nitrogen acquisition than nitrogen fixation. It has been shown that especially legumes induce high rhizosphere priming, potentially allowing them to first rely on mineral nitrogen and then switching to nitrogen fixation (Henneron et al., 2020). This strategy could be highly effective in combination with species that do not use the same strategy; however, in legume–legume interactions, it might lead to stronger competition since both species use the same strategy. This might be especially true when priority effects are present, since depletion of mineral nitrogen by the early‐arriving species might suppress rhizosphere priming by the later‐arriving species. Combined with the continued fast growth of the early‐arriving legume supported by nitrogen fixation, competition for light may further increase the competitive pressure on the late‐arriving legume. However, further studies on the underlying mechanisms of priority effects in legume–legume interactions are needed.

Previous studies show that legumes can cause positive priority effects (  Körner et al., 2008; Weidlich et al., 2018); however, we did not find any evidence for this. Those effects likely only play out over longer time spans, such as observed by (Delory, Weidlich, von Gillhaussen, et al., 2019), who found that priority effects by legumes became positive after 2 years. More in line with our findings, an experiment using the invasive plant species (*Senecio inaequidens*) found that late‐arriving groups of species were less impacted by priority effects by the invasive species if the group included legumes (Delory, Weidlich, Kunz, et al., 2019). Hence, the nitrogen‐fixing ability of legumes not only has the potential to create positive priority effects when arriving early but it might also make legumes less sensitive to priority effects when arriving late.

### Plant functional traits

In support of our third hypothesis, plant functional traits associated with an acquisitive–conservative gradient of both the focal and neighboring species explained a small but significant part of the variation in competition outcomes when species arrived simultaneously. Further, when species arrived sequentially (i.e., with priority effects present), these traits were able to explain much more variation. This shows that priority effects not only intensify competition between species but also make the plant functional traits of the early‐ and late‐arriving species more important. The shift in competitive hierarchy when priority effects are present that we observed when correlating competitive effects and responses can therefore partly be explained by a change in the importance of plant functional traits in determining the outcome of competition.

In support of our final hypothesis, we found that trait dissimilarities (i.e., the difference in PC1 scores of early and late‐arriving species) can predict the competitive outcome of interacting species. When species arrived simultaneously, the model was significant but with little predictive power. However, when species arrived sequentially, the trait dissimilarity explained a substantial amount of the variation in RII_seq_. This again shows that traits become more important when priority effects are present, but specifically, the *interaction* between traits of early‐ and late‐arriving species becomes important. Interestingly, we observed a threshold: The late‐arriving species had to be more acquisitive than the early‐arriving species in order for priority effects to become weaker. When the species have similar strategies, or the early‐arriving species is more acquisitive, priority effects are large to an extent of almost completely outcompeting the late‐arriving species.

Our PC1 axis corresponded to the leaf economic spectrum (LES) which is a commonly observed trait gradient (Diaz et al., 2004; Wang et al., 2019; Wright et al., 2004; Zhang et al., 2017) that has been used to explain ecological strategies (Zhang et al., 2017), flood resilience (Oram et al., 2020), and decomposition rates (de la Riva et al., 2019). A similar spectrum of traits has also been used to explain species interactions (Kraft et al., 2015; Liu et al., 2017), as we have performed here.

Previous studies have used trait differences to explain both coexistence and competitive exclusion (e.g., Angert et al., 2009; Kraft et al., 2015; Stubbs & Bastow Wilson, 2004). For example, Herben et al. (2020) demonstrated significant correlations between differences in mean ramet biomass, leaf area, and rooting depth, and competition strength across 19 grassland species. Further, Conti et al. (2018) used Euclidean distances between height, SLA, and seed mass to explain biotic resistance of native species to invading species. The effect of differences in root traits on competition across four grasses was studied by Fort et al. (2014), who found that differences in trait scores of grasses could predict competition intensity. However, as far as we are aware, we are the first to show that trait dissimilarities might be more important when priority effects are present.

Herben and Goldberg (2014) concluded that dissimilarity in growth traits led to differences in competitive ability, thereby reducing community diversity, whereas dissimilarity of architectural traits had either a positive or no effect on community diversity. Our finding aligns with these results, since growth traits (PC1) affected competition outcomes, while architectural traits (PC2) did not. Since this is a short‐term experiment on two‐species interactions, we cannot extrapolate to effects on community diversity, but this result does indicate that dissimilarity in competitive traits could lead to competitive exclusion. Further, dissimilarity in competitive traits becomes even more important when priority effects are present; however, in this case, dissimilarity can actually lead to a higher chance of coexistence. Specifically, coexistence is more likely when the late‐arriving species is more competitive than the early‐arriving species.

Since species are differently adapted, the competitive hierarchy in our species set would likely be different in other environments. For example, nutrient‐rich potting soil and ample water availability likely favored *Phalaris arundinacea*, which was indeed the strongest competitor when species arrived simultaneously. Had we used nutrient‐poor soil and less frequent watering, other species such as *Antennaria dioica* and *Sedum acre* might have been more competitive. Therefore, further research on the robustness of our results under varying environmental conditions and in other species sets would provide valuable insights. Additional studies on how priority effects shift competitive hierarchies to favor legumes and the underlying mechanisms of priority effects in legume–legume interactions would be particularly interesting. Further, we solely measured plant functional traits on the control plants; however, plant functional traits are plastic and can change due to both abiotic and biotic factors (Abakumova et al., 2016). Research on how plant functional traits themselves are affected by competition and priority effects would be interesting.

Finally, our experiment was conducted in a controlled environment over a relatively short time span, making it speculative to discuss how these relationships would hold up over larger temporal and spatial scales. For example, it has been shown that plant functional traits are decent predictors of ecosystem properties within a given year, but are poor predictors across multiple years (der Plas et al., 2020). We suspect that traits related to competitive ability are important during early establishment, but that the usefulness of certain traits and their values may shift as the environment changes, such as through plant–soil feedback (Kardol et al., 2007) or changes in weather or climate (der Plas et al., 2020). However, in systems dominated by perennials, this early establishment phase is likely important and several studies have demonstrated that early‐arriving species can remain dominant for multiple years (Vaughn & Young, 2015; Werner et al., 2016). This may be due to priority effects, which lead to asymmetric competition between early‐ and late‐arriving species, allowing early‐arriving species to maintain a competitive advantage, even when the late‐arriving species possess plant functional traits better suited for the changed environment.

In conclusion, our study gained important mechanistic insights into the role of plant functional traits as drivers of priority effects that may also impact long‐term plant community assembly and diversity. Understanding why certain species have a competitive advantage and how competition is affected by priority effects has applications in ecological restoration (Weidlich et al., 2018) and invasion ecology (Dickson et al., 2012). In our study, we show that priority effects do not simply make strong competitors stronger when they arrive early, but that the competitive hierarchy changes due to priority effects and that traits mediate competition outcomes differently when priority effects are present.

## CONFLICT OF INTEREST STATEMENT

The authors declare no conflicts of interest.

## Supporting information


Appendix S1:

## Data Availability

Data for the alone, simultaneous arrival, and second arrival treatments (van Steijn, 2024a) are available in Zenodo at https://doi.org/10.5281/zenodo.10512662. Trait value data from individuals grown alone and the code file for this work (van Steijn, 2024b) are available in Zenodo at https://doi.org/10.5281/zenodo.13849675.
