# Supplementary material for: Priority effects can be explained by competitive traits
Source: Ecology. 2025 Jan 21;106(1):e4528. doi: 10.1002/ecy.4528 (PMC11751378; doi:10.1002/ecy.4528)
Supplement: Supplementary file 1 — Appendix S1: [file ECY-106-e4528-s001.pdf]

Priority effects can be explained by competitive traits

Tamara L.H. van Steijn, Paul Kardol, Roland Jansson, Jessica Tjäder,
and Judith M. Sarneel

Ecology

Contents

Appendix S1

S1.1 Soil analysis 2
S1.2 Picture of set-up 3
S1.3 Biomass 4
S1.4 Competitive effect and response 5
S1.5 ANOVA of RII above- and below-ground and plant functional groups 6
S1.6 LME of PC1 traits 11
S1.7 LME of PC2 traits 13

Appendix S1

S1.1 Soil analysis

Soil extracts were made by shaking 5 g of the soil mixture in 25 ml of water followed by filtration using 0.45 µm Filtropur.

The TOC (or DOC) is measured by combustion (870 ºC) of acidified water samples (bubbled with O2), and then analyzed with an infrared gas analyzer (IRGA). The samples are analyzed on a Formacs HT-I from Skalar. TN (or TDN) is analyzed on an ND25 unit connected to the Fomacs using a chemiluminescent detector. TC is analyzed on non-acidified water samples, while IC samples are injected into a chamber containing 2% H3PO4 before the samples are combusted and analyzed on the IRGA. Particulate carbon is analyzed by combustion (1100 ºC) of the solid sample using a Primacs module to the Formacs. The sample is after combustion analyzed on the infrared gas analyzer on the Formacs.

Dissolved and total nutrients is analyzed by measuring color on a photometer in a segmented flow analyzer, after various reagents have been a dded. NO3 (NO3+NO2) after reagents and samples passed a copperized Cd reduction coil to form an azo dye [method: MT3B Q-126-12 Rev 1]. NH4 with the salicylate method [method: Q-033-04 Rev. 8] and PO4 with the molybdenum blue method [method: MT3A Q-125-12 Rev 1].

Total nutrients analyzed in the same manner after having passed an on-line digestion step (alkaline acidic persulfate method) at 110 ºC and 0.9 MPa [method: Q-115-10 Rev. 4]. Nutrients have been analyzed on a QuAAtro 39 from Seal Analytical.

CO2 and CH4 are analyzed on a Clarus 500 gas chromatograph (Perkin Elmer). Samples are preheated to a constant temperature (40 ºC) on the headspace sampler before injection to the capillary column (Elite-Plot Q, 30m, 0.53mm ID). Oven temperature is set to 100 ºC, and the detector temperature to 250 ºC. The samples are analyzed on a FID, equipped with a methanizer. N2O samples are analyzed on the GC in a separate injector port connected to an ECD (at 375 ºC). The detector is flushed with make-up gas (P5, Ar with 5% CH4). The column is a capillary Q-Plot column, and the oven temperature is set to 100 ºC. Carrier gas is N2 for all analyses. For results see table S1.

Table S1: Soil analysis of the soil mixture used in the experiment.

Soil sample	pH	NO3_N mg/L	NH4-N mg/L	PO4-P mg/L
1	6.82	3.4	15.1	5.5
2	6.89	3	13.3	5.1
3	6.8	4	17.7	6.7
4	6.91	3.9	18	7.3
5	6.73	4.9	23.8	8.2
Mean	6.83	3.84	17.58	6.56
SD	0.072457	0.71624	3.979573	1.275931

S1.2 Picture of set-up

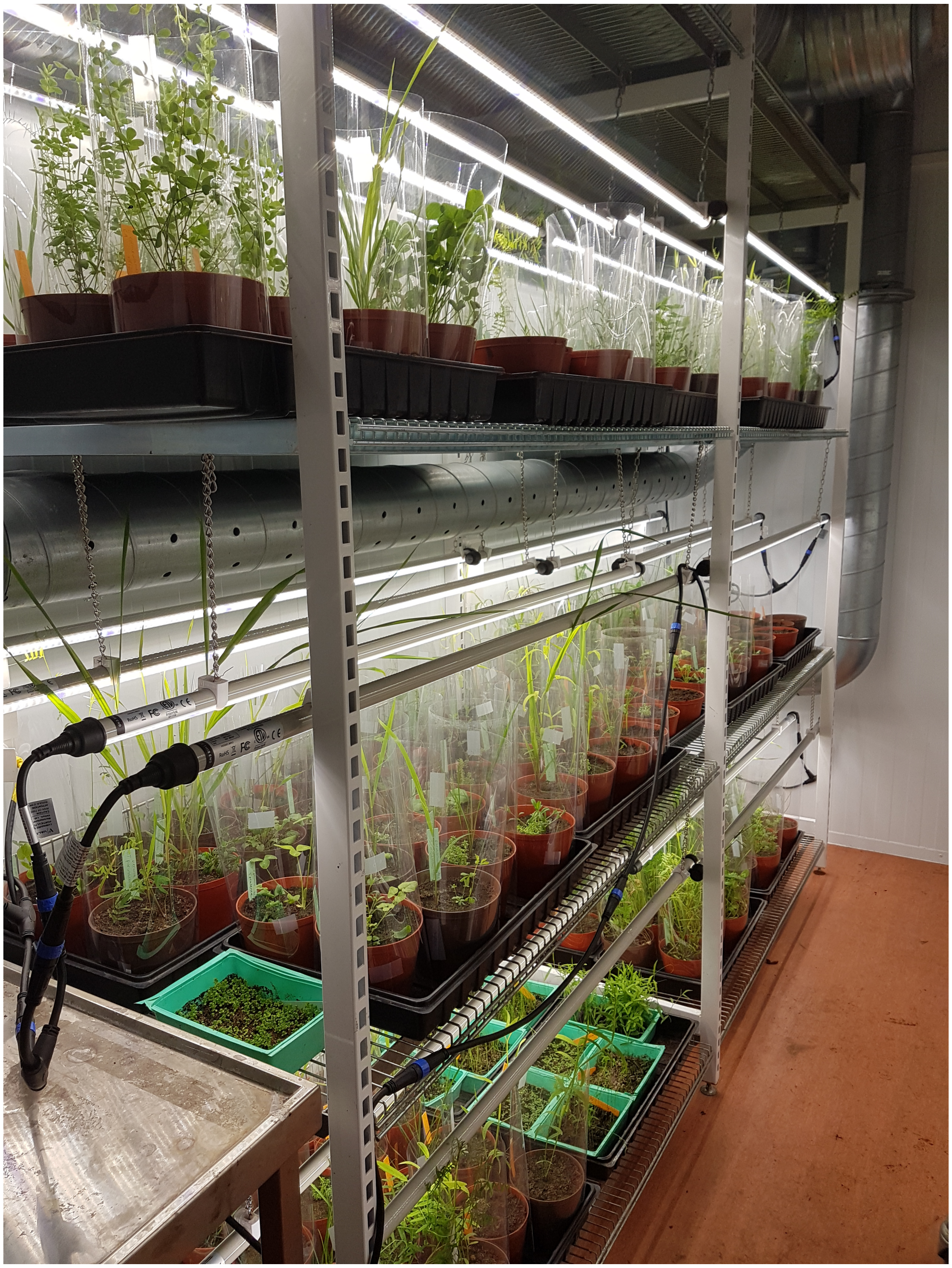

Figure S1: Picture of the set-up mid-experiment, showing the plants competing in pots wrapped in transparent cones.

S1.3 Biomass

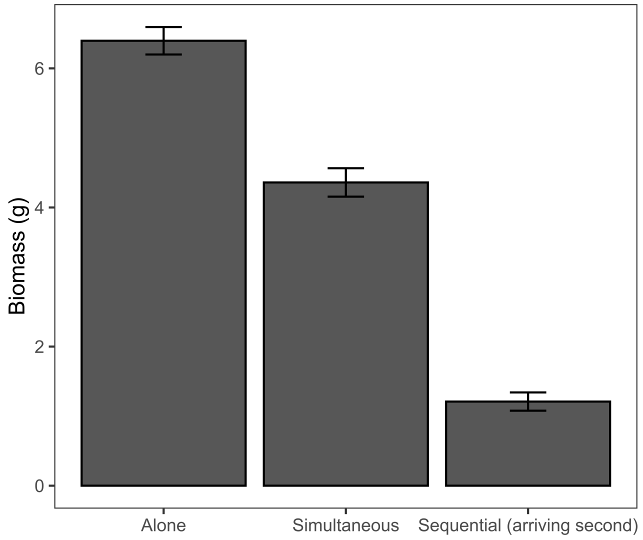

Figure S2: Average biomasses for plants grown alone, when arriving simultaneously and when arriving second. Standard error bars are displayed. n = 75 for alone (5 replicates for 15 species), 210 for sequential (each of 15 species arriving late for each of 14 possible early arrivals), and 210 for simultaneous (each of 15 species growing together with each of 14 possible neighbors).

S1.4 Competitive effect and response

Table S2: Linear regression model summary with competitive effect as the dependent variable and competitive response as the independent variable for RII_{sim}.

	Df	Sum Sq	Mean Sq	F value	Pr(>F)
response	1	0.16	0.16	49.67	<**0.001**
Residuals	13	0.04	0.00		
Multiple r-squared = 0.79, Adj. r-squared = 0.78, p-value = <**0.001**					

Table S3: Linear regression model summary with competitive effect as the dependent variable and competitive response as the independent variable for RII_{seq}.

	Df	Sum Sq	Mean Sq	F value	Pr(>F)
response	1	0.16	0.16	15.32	**0.0021**
$response^2$	1	0.14	0.14	13.37	**0.0033**
Residuals	12	0.13	0.01		
Multiple r-squared = 0.71, Adj. r-squared = 0.66, p-value = <**0.001**					

S1.5 ANOVA of RII above- and below-ground and plant functional groups

Table S4: Three-way ANOVA of the effect of competition space (above-or-below) and plant functional group (PFG) of the focal and neighboring species on the Relative Interaction Indices of simultaneous (RII_{sim}) or sequential (RII_{seq}) arrival order (Fig. 4).

	Df	Sum Sq	Mean Sq	F value	Pr(>F)	TukeyHSD
**RII_{sim}**						
above- or below-ground (ABG)	1	0.38	0.38	5.68	**0.018**	
PFG focal	2	3.29	1.65	24.53	<**0.001**	table S5
PFG neighbor	2	0.42	0.21	3.15	**0.044**	table S6
ABG x PFG focal	2	0.84	0.42	6.29	**0.002**	table S7
ABG x PFG neighbor	2	0.26	0.13	1.91	0.150	
PFG focal x PFG neighbor	4	0.63	0.16	2.36	**0.053**	table S8
ABG x PFG focal x PFG neighbor	4	0.16	0.04	0.59	0.673	
Residuals	394	26.44	0.07			
**RII_{seq}**						
ABG	1	0.00	0.00	0.04	0.838	
PFG focal	2	9.89	4.94	81.99	<**0.001**	table S5
PFG neighbor	2	2.04	1.02	16.91	<**0.001**	table S6
ABG x PFG focal	2	0.01	0.00	0.07	0.933	
ABG x PFG neighbor	2	0.05	0.02	0.38	0.682	
PFG focal x PFG neighbor	4	1.70	0.43	7.06	<**0.001**	table S9
ABG x PFG focal x PFG neighbor	4	0.20	0.05	0.82	0.511	
Residuals	386	23.27	0.06			

Table S5: Tukey-HSD output for the effect of the plant functional group (PFG) of the focal species on RII_{sim} and RII_{seq}. *diff* gives the differences between the observed means, *lwr* and *upr* give the lower and upper end point of the interval, respectively.

$\mathbf{RII_{sim}}$	diff	lwr	upr
Grass-Forb	0.16	0.09	0.24
Legume-Forb	0.21	0.13	0.28
Legume-Grass	0.05	-0.03	0.12
$\mathbf{RII_{seq}}$			
Grass-Forb	-0.02	-0.09	0.05
Legume-Forb	0.32	0.25	0.39
Legume-Grass	0.34	0.27	0.41

Table S6: Tukey-HSD output for the effect of the plant functional group (PFG) of the neighboring species on RII_{sim} and RII_{seq}. *diff* gives the differences between the observed means, *lwr* and *upr* give the lower and upper end point of the interval, respectively.

$\mathbf{RII_{sim}}$	diff	lwr	upr
Grass-Forb	-0.04	-0.11	0.04
Legume-Forb	-0.08	-0.15	-0.00
Legume-Grass	-0.04	-0.11	0.03
$\mathbf{RII_{seq}}$			
Grass-Forb	-0.07	-0.14	0.00
Legume-Forb	-0.17	-0.24	-0.10
Legume-Grass	-0.10	-0.17	-0.03

Table S7: Tukey-HSD output of the interaction between competition space (above-or-below) and the plant functional group of the focal species (ABG x PFG focal). *diff* gives the differences between the observed means, *lwr* and *upr* give the lower and upper end point of the interval, respectively.

$\mathbf{RII_{sim}}$	diff	lwr	upr
below:Forb-above:Forb	-0.12	-0.25	0.01
above:Grass-above:Forb	0.17	0.04	0.29
below:Grass-above:Forb	0.04	-0.09	0.16
above:Legume-above:Forb	0.11	-0.01	0.24
below:Legume-above:Forb	0.18	0.05	0.31
above:Grass-below:Forb	0.29	0.16	0.41
below:Grass-below:Forb	0.16	0.03	0.28
above:Legume-below:Forb	0.24	0.11	0.36
below:Legume-below:Forb	0.30	0.17	0.43
below:Grass-above:Grass	-0.13	-0.26	-0.00
above:Legume-above:Grass	-0.05	-0.18	0.08
below:Legume-above:Grass	0.01	-0.11	0.14
above:Legume-below:Grass	0.08	-0.05	0.21
below:Legume-below:Grass	0.14	0.02	0.27
below:Legume-above:Legume	0.07	-0.06	0.19

Table S8: Tukey-HSD output of the interaction between the plant functional group of the focal species and the plant functional group of the neighboring species (PFG focal x PFG neighbor). *diff* gives the differences between the observed means, *lwr* and *upr* give the lower and upper end point of the interval, respectively.

**RII_{sim}**	diff	lwr	upr
Grass:Forb-Forb:Forb	0.14	-0.03	0.32
Legume:Forb-Forb:Forb	0.28	0.11	0.45
Forb:Grass-Forb:Forb	0.02	-0.15	0.19
Grass:Grass-Forb:Forb	0.11	-0.08	0.30
Legume:Grass-Forb:Forb	0.18	0.01	0.35
Forb:Legume-Forb:Forb	-0.07	-0.24	0.10
Grass:Legume-Forb:Forb	0.16	-0.01	0.33
Legume:Legume-Forb:Forb	0.09	-0.09	0.27
Legume:Forb-Grass:Forb	0.13	-0.03	0.30
Forb:Grass-Grass:Forb	-0.13	-0.29	0.04
Grass:Grass-Grass:Forb	-0.03	-0.21	0.14
Legume:Grass-Grass:Forb	0.03	-0.13	0.20
Forb:Legume-Grass:Forb	-0.22	-0.38	-0.05
Grass:Legume-Grass:Forb	0.02	-0.15	0.18
Legume:Legume-Grass:Forb	-0.05	-0.23	0.12
Forb:Grass-Legume:Forb	-0.26	-0.42	-0.09
Grass:Grass-Legume:Forb	-0.17	-0.34	0.01
Legume:Grass-Legume:Forb	-0.10	-0.26	0.06
Forb:Legume-Legume:Forb	-0.35	-0.51	-0.19
Grass:Legume-Legume:Forb	-0.11	-0.28	0.05
Legume:Legume-Legume:Forb	-0.19	-0.36	-0.01
Grass:Grass-Forb:Grass	0.09	-0.09	0.27
Legume:Grass-Forb:Grass	0.16	-0.01	0.32
Forb:Legume-Forb:Grass	-0.09	-0.25	0.07
Grass:Legume-Forb:Grass	0.14	-0.02	0.31
Legume:Legume-Forb:Grass	0.07	-0.10	0.25
Legume:Grass-Grass:Grass	0.07	-0.11	0.24
Forb:Legume-Grass:Grass	-0.18	-0.36	-0.01
Grass:Legume-Grass:Grass	0.05	-0.12	0.23
Legume:Legume-Grass:Grass	-0.02	-0.20	0.17
Forb:Legume-Legume:Grass	-0.25	-0.41	-0.09
Grass:Legume-Legume:Grass	-0.01	-0.18	0.15
Legume:Legume-Legume:Grass	-0.09	-0.26	0.09
Grass:Legume-Forb:Legume	0.23	0.07	0.40
Legume:Legume-Forb:Legume	0.16	-0.01	0.34
Legume:Legume-Grass:Legume	-0.07	-0.24	0.10

Table S9: Tukey-HSD output of the interaction between the plant functional group of the focal species and the plant functional group of the neighboring species (PFG focal x PFG neighbor). *diff* gives the differences between the observed means, *lwr* and *upr* give the lower and upper end point of the interval, respectively.

$\mathbf{RII_{seq}}$	diff	lwr	upr
Grass:Forb-Forb:Forb	-0.03	-0.20	0.14
Legume:Forb-Forb:Forb	0.41	0.25	0.58
Forb:Grass-Forb:Forb	-0.07	-0.23	0.10
Grass:Grass-Forb:Forb	-0.06	-0.24	0.12
Legume:Grass-Forb:Forb	0.32	0.16	0.48
Forb:Legume-Forb:Forb	-0.07	-0.23	0.09
Grass:Legume-Forb:Forb	-0.10	-0.27	0.06
Legume:Legume-Forb:Forb	0.03	-0.14	0.20
Legume:Forb-Grass:Forb	0.45	0.29	0.60
Forb:Grass-Grass:Forb	-0.04	-0.20	0.12
Grass:Grass-Grass:Forb	-0.03	-0.20	0.15
Legume:Grass-Grass:Forb	0.35	0.19	0.51
Forb:Legume-Grass:Forb	-0.04	-0.20	0.12
Grass:Legume-Grass:Forb	-0.07	-0.23	0.08
Legume:Legume-Grass:Forb	0.06	-0.11	0.23
Forb:Grass-Legume:Forb	-0.48	-0.64	-0.33
Grass:Grass-Legume:Forb	-0.47	-0.64	-0.30
Legume:Grass-Legume:Forb	-0.10	-0.25	0.06
Forb:Legume-Legume:Forb	-0.49	-0.64	-0.33
Grass:Legume-Legume:Forb	-0.52	-0.67	-0.37
Legume:Legume-Legume:Forb	-0.38	-0.55	-0.22
Grass:Grass-Forb:Grass	0.01	-0.16	0.18
Legume:Grass-Forb:Grass	0.39	0.23	0.54
Forb:Legume-Forb:Grass	-0.00	-0.16	0.15
Grass:Legume-Forb:Grass	-0.04	-0.19	0.12
Legume:Legume-Forb:Grass	0.10	-0.07	0.26
Legume:Grass-Grass:Grass	0.38	0.21	0.55
Forb:Legume-Grass:Grass	-0.01	-0.18	0.16
Grass:Legume-Grass:Grass	-0.05	-0.22	0.12
Legume:Legume-Grass:Grass	0.09	-0.09	0.27
Forb:Legume-Legume:Grass	-0.39	-0.54	-0.24
Grass:Legume-Legume:Grass	-0.42	-0.58	-0.27
Legume:Legume-Legume:Grass	-0.29	-0.45	-0.13
Grass:Legume-Forb:Legume	-0.03	-0.19	0.12
Legume:Legume-Forb:Legume	0.10	-0.06	0.26
Legume:Legume-Grass:Legume	0.13	-0.03	0.30

S1.6 LME of PC1 traits

Table S10: Type III Analysis of variance table with Satterthwaite's method for the relative intensity index when species arrive simultaneously (RII_{sim}). Fixed effect included the PC1 values of the focal species, the PC1 values of the neighboring species, competition space (above- or below-ground) and all possible interactions. The random effects included species ID of the focal species, species ID of the neighboring species and the pot ID.

	Sum Sq	Mean Sq	NumDF	DenDF	F value	Pr(>F)
PC1 Focal (PF)	0.27	0.27	1	12.97	8.19	**0.013**
PC1 Neighbour (PN)	0.24	0.24	1	12.99	7.13	**0.019**
Above- or below-ground (ABG)	0.38	0.38	1	379.35	11.51	**0.001**
PF x ABG	0.60	0.60	1	379.35	17.92	<**0.001**
PN x ABG	0.11	0.11	1	379.35	3.19	0.075
PF x PN	0.04	0.04	1	380.18	1.19	0.276

Table S11: Type III Analysis of variance table with Satterthwaite's method for the relative intensity index when species arrive sequentially (RII_{seq}). Fixed effect included the PC1 values of the focal species, the PC1 values of the neighboring species, competition space (above- or below-ground) and all possible interactions. The random effects included species ID of the focal species, species ID of the neighboring species and the pot ID.

	Sum Sq	Mean Sq	NumDF	DenDF	F value	Pr(>F)
PC1 Focal (PF)	0.58	0.58	1	12.99	20.55	**0.001**
PC1 Neighbour (PN)	0.21	0.21	1	12.99	7.44	**0.017**
Above- or below-ground (ABG)	0.00	0.00	1	283.16	0.09	0.758
PF x ABG	0.00	0.00	1	283.16	0.06	0.811
PN x ABG	0.00	0.00	1	283.16	0.08	0.773
PF x PN	0.30	0.30	1	84.52	10.74	**0.002**

Table S12: Type III Analysis of variance table with Satterthwaite's method for the effect of trait dissimilarity on the relative intensity index (RII_{sim}) when species arrive simultaneously. Fixed effect included the difference in PC1 values, competition space (above- or below-ground) and their interaction. The random effects included species ID of the focal species, species ID of the neighboring species and the pot ID.

	Sum Sq	Mean Sq	NumDF	DenDF	F value	Pr(>F)
PC1 difference (PC1 diff)	0.46	0.46	1	20.15	13.61	**0.001**
Above- or- below-ground (ABG)	0.38	0.38	1	381.34	11.35	**0.001**
PC1 diff x ABG	0.65	0.65	1	381.34	19.39	<**0.001**

Table S13: Type III Analysis of variance table with Satterthwaite's method for the effect of trait dissimilarity on the relative intensity index (RII_{seq}) when species arrive sequentially. Fixed effect included the difference in PC1 values, competition space (above- or below-ground) and their interaction. The random effects included species ID of the focal species, species ID of the neighboring species and the pot ID.

	Sum Sq	Mean Sq	NumDF	DenDF	F value	Pr(>F)
PC1 difference^2 (PC1 diff)	1.24	0.62	2.00	37.06	22.09	<**0.001**
Above- or- below-ground (ABG)	0.00	0.00	1.00	282.98	0.09	0.7651
PC1 diff x ABG	0.01	0.00	2.00	282.98	0.09	0.9122

S1.7 LME of PC2 traits

Table S14: Type III Analysis of variance table with Satterthwaite's method the relative intensity index (RII_{sim}) when species arrive simultaneously. Fixed effect included the PC2 values of the focal species, the PC2 values of the neighboring species, competition space (above- or below-ground) and all possible interactions. The random effects included species ID of the focal species, species ID of the neighboring species and the pot ID.

	Sum Sq	Mean Sq	NumDF	DenDF	F value	Pr(>F)
PC2 Focal (PN)	0.06	0.06	1	12.97	1.80	0.202
PC2 Neighbour (PN)	0.01	0.01	1	12.93	0.16	0.694
Above- or below-ground (ABG)	0.38	0.38	1	379.19	10.98	**0.001**
PF x ABG	0.13	0.13	1	379.19	3.60	0.0584
PN x ABG	0.02	0.02	1	379.19	0.53	0.4685
PF x PN	0.10	0.10	1	380.33	2.89	0.0899

Table S15: Type III Analysis of variance table with Satterthwaite's method the relative intensity index (RII_{seq}) when species arrive sequentially. Fixed effect included the PC2 values of the focal species, the PC2 values of the neighboring species, competition space (above- or below-ground) and all possible interactions. The random effects included species ID of the focal species, species ID of the neighboring species and the pot ID.

	Sum Sq	Mean Sq	NumDF	DenDF	F value	Pr(>F)
PC2 Focal (PN)	0.01	0.01	1	13.02	0.52	0.482
PC2 Neighbour (PN)	0.03	0.03	1	12.95	1.21	0.292
Above- or below-ground (ABG)	0.00	0.00	1	282.97	0.07	0.790
PF x ABG	0.03	0.03	1	282.97	1.14	0.287
PF x ABG	0.26	0.26	1	282.97	9.71	**0.002**
PF x PN	0.00	0.00	1	87.13	0.01	0.920

Table S16: Type III Analysis of variance table with Satterthwaite's method for the effect of trait dissimilarity on the relative intensity index (RII_{sim}) when species arrive simultaneously. Fixed effect included the difference in PC2 values, competition space (above- or below-ground) and their interaction. The random effects included species ID of the focal species, species ID of the neighboring species and the pot ID.

	Sum Sq	Mean Sq	NumDF	DenDF	F value	Pr(>F)
PC2 difference (PC2 diff)	0.04	0.04	1	20.74	1.04	0.319
Above- or below-ground (ABG)	0.38	0.38	1	381.20	10.90	**0.001**
PC2 diff x ABG	0.13	0.13	1	381.20	3.71	0.0547

Table S17: Type III Analysis of variance table with Satterthwaite's method for the effect of trait dissimilarity on the relative intensity index (RII_{seq}) when species arrive simultaneously. Fixed effect included the difference in PC2 values, competition space (above- or below-ground) and their interaction. The random effects included species ID of the focal species, species ID of the neighboring species and the pot ID.

	Sum Sq	Mean Sq	NumDF	DenDF	F value	Pr(>F)
PC2 difference (PC2 diff)	0.00	0.00	1	26.13	0.09	0.768
Above- or below-ground (ABG)	0.00	0.00	1	283.98	0.09	0.758
PC2 diff x ABG	0.06	0.06	1	283.98	2.22	0.137

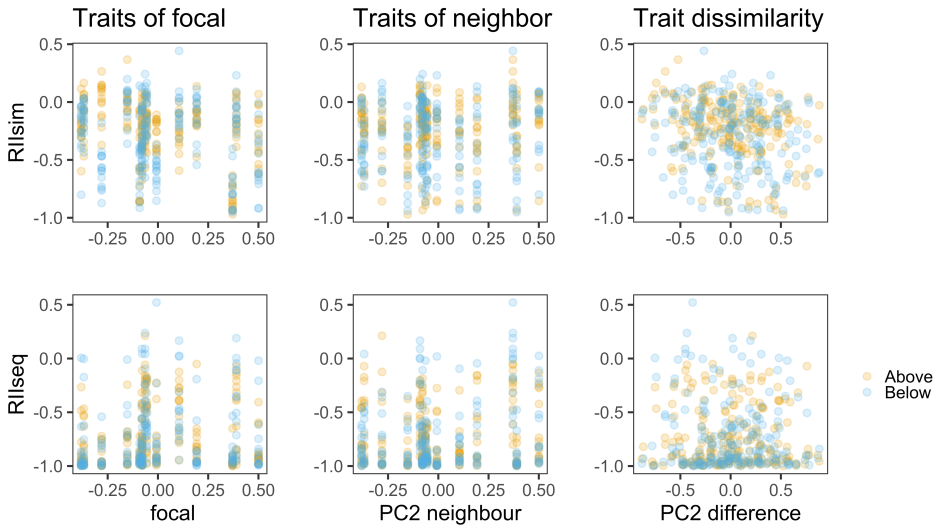

Figure S3: Relationships between PC2 species values and the Relative Intensity Indices, which are calculated based on above- and below-ground for RII_{sim} and RII_{seq}. RII values of around zero indicate no effect of competition, while values close to -1 indicate strong competition. Each dot is one pot.
